# Improved distinct bone segmentation from upper-body CT using binary-prediction-enhanced multi-class inference.

**DOI:** 10.1007/s11548-022-02650-y

**Published:** 2022-05-20

**Authors:** Eva Schnider, Antal Huck, Mireille Toranelli, Georg Rauter, Magdalena Müller-Gerbl, Philippe C. Cattin

**Affiliations:** 1 Department of Biomedical Engineering, University of Basel, Gewerbestrasse 14, Allschwil, 4123 Switzerland; 2 Department of Biomedicine, Musculoskeletal Research, University of Basel, Basel, Switzerland

**Keywords:** U-Net, Deep-learning, Distinct bone segmentation, CT

## Abstract

****Purpose**:**

Automated distinct bone segmentation has many applications in planning and navigation tasks. 3D U-Nets have previously been used to segment distinct bones in the upper body, but their performance is not yet optimal. Their most substantial source of error lies not in confusing one bone for another, but in confusing background with bone-tissue.

****Methods**:**

In this work, we propose binary-prediction-enhanced multi-class (BEM) inference, which takes into account an additional binary background/bone-tissue prediction, to improve the multi-class distinct bone segmentation. We evaluate the method using different ways of obtaining the binary prediction, contrasting a two-stage approach to four networks with two segmentation heads. We perform our experiments on two datasets: An in-house dataset comprising 16 upper-body CT scans with voxelwise labelling into 126 distinct classes, and a public dataset containing 50 synthetic CT scans, with 41 different classes.

****Results**:**

The most successful network with two segmentation heads achieves a class-median Dice coefficient of 0.85 on cross-validation with the upper-body CT dataset. These results outperform both our previously published 3D U-Net baseline with standard inference, and previously reported results from other groups. On the synthetic dataset, we also obtain improved results when using BEM-inference.

****Conclusion**:**

Using a binary bone-tissue/background prediction as guidance during inference improves distinct bone segmentation from upper-body CT scans and from the synthetic dataset. The results are robust to multiple ways of obtaining the bone-tissue segmentation and hold for the two-stage approach as well as for networks with two segmentation heads.

## Introduction

The segmentation of various distinct bones visible on CT scans is a powerful way to provide semantic information and feedback to planning and navigation tools [1]. Bone segmentations can also be used as a strong starting point for atlas-based approaches [2], or as location anchors to detect organs and other body structures [3]. Bone segmentation has also sparked interest as a possible alternative or add-on to augmented reality visualization of medical data and intraoperative workspaces [4].

**Fig. 1:**
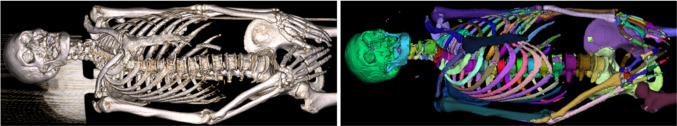
Volume rendering of one of our upper-body CT scans (left), and the result of our automated segmentation using BEM-inference and label-correction (right)

Manual segmentation requires a trained medical professional to go through an image slice by slice and mark voxels as part of the structure of interest. This approach is time-consuming and hard to scale up. Interactive segmentation tools help by offering automated steps such as thresholding and morphological operations to decrease the time needed for (semi-)manual segmentation. For bone-tissue segmentation from CT, convolutional neural networks (CNN) have been found to clearly outperform threshold-based approaches [5, 6].

In contrast to bone-tissue segmentation, which aims at differentiating between the background and bone-tissue in general, distinct bone segmentation also separates one bone from another. The task is well-studied for vertebrae segmentation, but the reliance on the sequential nature of the spine hinders a direct adoption to other body parts [7]. A total of five bones in the ankle and shoulder region are segmented in [8], where they use a U-Net [9, 10] in combination with shape priors and adversarial regularization. They also compare the performance of separate U-Nets trained on one bone class each versus a multi-class U-Net which outperformed the combined single-class networks.

Segmentation into a larger number of distinct bones has not yet been investigated in many cases. A hierarchical atlas-based approach leads to good segmentation results of 62 distinct bones from upper-body CTs at the expense of a long inference time [2]. In [11], 49 distinct bone classes have been segmented on upper-body CTs. They used a two-stage approach where a landmark detection network was followed by a voxelwise segmentation by a dilation-based CNN and the deletion of all but the largest connected component per class. Neither of these two approaches offers an end-to-end method or includes the bones of the hand in the segmentation. A segmentation that also includes these bones, totalling to 126 bone classes, has been investigated on a smaller dataset in one of our previous works [12], where we found a 3D U-Net to be better suited to the task than the 2D U-Nets commonly used in a slicewise way for bone-tissue segmentation.

The purpose of of this current work is to reduce the most prevalent segmentation errors of the 3D U-Net when performing distinct bone segmentation. To do so, we propose to leverage an additional binary segmentation during the inference process. A related approach has been examined by [13] who combine the outputs of a semantic segmentation head and an instance segmentation head into a panoptic segmentation for 2D traffic images. Apart from the dimensionality and the image modality, our work also differs as we stay within a semantic segmentation problem statement.

We propose and investigate BEM, an inference method that enhances a multi-class distinct bone segmentation using a binary bone-tissue/background segmentation. We compare the segmentation accuracy, run-time, and complexity of different network architectures that achieve both segmentations within a single trained model, and contrast the results to a two-stage approach.

## Materials and methods

### Upper-body CT dataset

Our in-house dataset consists of 17 upper-body CT scans, and corresponding voxelwise segmentations created by specialists, with an isotropic resolution of 2 mm , as used in [14]. The dataset comprises postmortem scans of 9 male and 7 female body donours aged 44–103 years. Before resampling, the scans were of varying resolution with slightly less than 1 mm resolution in-plane and up to 1.5 mm out-plane. Due to inconsistent arm positioning, we excluded one scan from the set in this work. The segmentation contains 126 different classes, including background (Fig. 1).

**Fig. 2:**
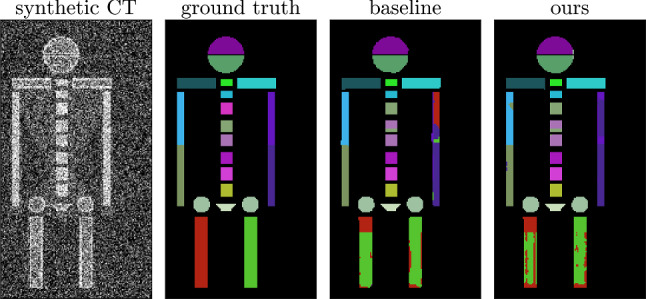
Results on the synthetic dataset using the baseline 3D U-Net, and Dual D with our proposed BEM-inference. Both false positives (around the elbows), and false negatives (head) are reduced using our approach

**Table 1:** Network architectures comparison for the upper-body CT dataset

Model	Trainable parameters (#)	Training time $^{1}$(s)	Inference time $^{2}$ (s)
Baseline 3D U-Net	$1.46\cdot 10^7$	0.84	219
Dual A	$1.46\cdot 10^7$	1.08	212
Dual B	$1.46\cdot 10^7$	1.08	271
Dual C	$1.46\cdot 10^7$	1.15	243
Dual D	$1.98\cdot 10^7$	1.20	321

$^{1}$Average time per training iteration on a $64^3$ voxel patch.

$^{2}$ Inference time for an average scan ($\sim 256\times 256\times 512$ voxels) , including data I/O time

**Fig. 3:**
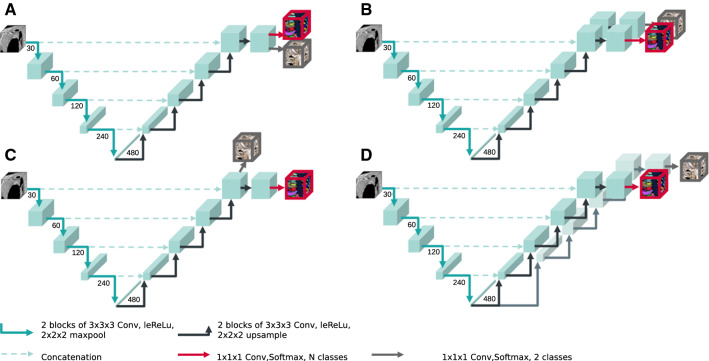
Schematic of the four network architectures with dual segmentation heads. They are all based on a 3D U-Net architectures with variances of how the binary segmentation head is appended. See also “Dual segmentation head architecture” Section

### Synthetic 3D dataset

We created a synthetic dataset in order to highlight the effect of the proposed BEM-inference on anatomical segmentation tasks and to provide results on a publicly available dataset (published at https://gitlab.com/cian.unibas.ch/cars2022-bem-inference). The dataset was constructed by generating a randomly varying three-dimensional stick-figure-like ground truth segmentation consisting of 41 distinct bones (see Fig. 2). Inspired by human anatomy, we chose similar geometric shapes for similar bones such as vertebrae, to force the networks to rely not only on shapes but also the relative positioning of structures. To construct the soft-tissue area, we created convex hulls for the torso, limbs, and head. Finally, we filled areas of background, soft-tissue, cortical bone and cancellous bone with typical HU-values and added uniform random noise. Emphasis is not put on the anatomical accuracy of the dataset, but on the ability to mimic the difficulty of our primary task, which is to study the simultaneous detection and distinction of many three-dimensional structures with groupwise similar shapes.. The final synthetic CT scans measure $128 \times 128 \times 256$ voxels.

### Base architecture

We use an architecture based on the 3D U-Net [10], which is composed of a decoder and encoder with skip connections. Following [15], we add instance normalization, use leaky rectified linear units (leReLU) and exchange the upconvolutions in favor of linear upsampling. The high computational demand of a 3D network with a large number of classes, restricts the possible batch size to one. We implemented the network in Tensorflow-Keras 2.5.

### Dual segmentation head architecture

To obtain the multi-class and the binary background/bone-tissue segmentation simultaneously, we explore four architectures with two segmentation heads. A comparison of their architectures is given in Table 1 and Fig. 3.

- **Dual A** All layers except the classification heads are shared.
- **Dual B** Both tasks still share the whole encoder and decoder but have their own convolutional layers at full resolution.
- **Dual C** Both tasks share the full encoder and decoder. The binary segmentation head is appended after the decoder, the distinct bone segmentation head follows after one more convolutional block at full resolution.
- **Dual D** Both tasks share the encoder and feature encoding, but have their own decoders.

**Fig. 4:**
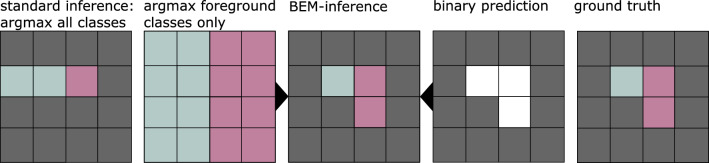
Schematic of the BEM-inference process. The background class is denoted in gray, the two distinct foreground classes in blue and pink, respectively

### Two-stage approach

As an alternative to the architectures with dual segmentation heads, we study the results using a binary prediction, which is obtained separately from the full multi-class network. To do so, we train an additional instance of our baseline 3D U-Net on the background/bone-tissue problem alone and use the resulting binary segmentation during the BEM-inference step. As an upper bound, we also compute results using the ground truth of the binary segmentation.

### Training and standard inference

For both datasets, we optimize our networks using the Adam optimizer with a learning rate of 0.001 for 75000 iterations, after which all of our models had converged. Total training time is roughly one day per cross-validation fold on one GeForce GTX Titan X (12 GB). We use five cross-validation splits for the upper-body dataset, where we use 11 scans for training, 2 for validation of the convergence, and 3 for testing. For the synthetic dataset, we were able to create a larger number of validation and test images to get more representative test results and thus evaluate one fold only. We use 17 volumes for training, 7 for validation, and 26 for testing.

As loss function we use an unweighted combination of the cross-entropy loss ${\mathcal {L}}_{\text {X-Ent}}$ and the Dice loss ${\mathcal {L}}_{\mathrm {DSC}}$ [16]. In the dual segmentation head networks, we add the losses for the binary background/bone-tissue task:

$$\begin{aligned} {\mathcal {L}}_{\text {total}} :={\mathcal {L}}^{C}_{\text {X-Ent}} + \sum _{c \in C} {\mathcal {L}}_{\mathrm {DSC}}^{c} + {\mathcal {L}}^{\left\{ \text {bg},\text {bt} \right\} }_{\text {X-Ent}} + \sum _{c \in \left\{ \text {bg},\text {bt} \right\} } {\mathcal {L}}_{\mathrm {DSC}}^{c} \end{aligned}$$

We train our network patchwise since the use of whole CT volumes for training is not computationally feasible in 3D. The patch size not only influences the computational requirements, but also the network accuracy [17]. We found a patch size of $64^3$ voxels to be a good compromise. The patchwise sampling also serves as a random-cropping data-augmentation step. Other common data augmentation techniques such as rotations, scaling, or mix-up are not used in this work. Data augmentation has been studied in-depth for whole-body bone-tissue segmentation, where it only leads to very small improvements [5].

Prior to inference, we pad our scans by 20 voxels to mitigate the proximity of the hands to the image border in some of the scans. After padding, our predictions are assembled using a sliding window approach with a 20 voxel overlap to increase the influence of the centre of the patches on the final predictions, which has been shown to lead to good results [15]. The voxelwise multi-class prediction is conducted by a softmax activation.

**Fig. 5:**
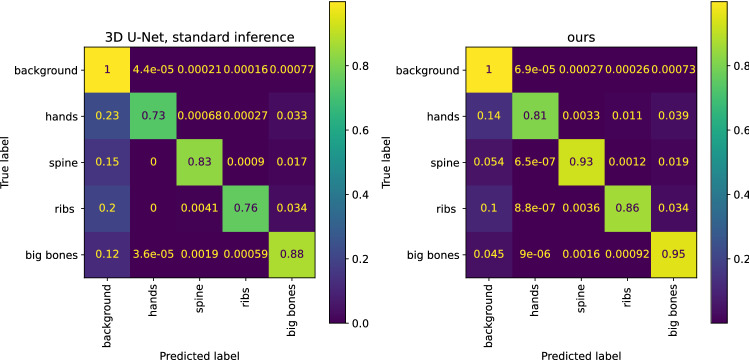
Label confusion matrices (row-normalized) for the baseline 3D U-Net and Dual D, including BEM-inference and post-processing. With our approach, less labels are erroneously classified as background (first column)

**Table 2:** Upper-body CT dataset: Results in DSC, comparing the segmentation performance when using baseline inference, against our BEM-inference, with and without label correction

	Baseline	+ Label correction	+ BEM-inference	+ Both
Baseline 3D U-Net	$0.78_{-0.29}^{+0.12}, (0.95)$	$0.81_{-0.25}^{+0.09}, (0.94)$		
Two-stage: pred. bin.	”	”	$0.79_{-0.30}^{+0.11}, (0.96)$	$0.82_{-0.26}^{+0.09}, (0.94)$
Two-stage: gt bin.	”	”	$0.89_{-0.29}^{+0.08}, (0.96)$	$0.93_{-0.22}^{+0.05}, (0.95)$
Dual A	$0.78_{-0.29}^{+0.11}, (0.96)$	$0.81_{-0.27}^{+0.09}, (0.95)$	$0.79_{-0.30}^{+0.11}, (0.97)$	$0.82_{-0.27}^{+0.10}, (0.95)$
Dual B	$0.77_{-0.28}^{+0.12}, (0.95)$	$0.81_{-0.29}^{+0.09}, (0.94)$	$0.79_{-0.30}^{+0.11}, (0.96)$	$0.82_{-0.28}^{+0.09}, (0.95)$
Dual C	$0.79_{-0.31}^{+0.10}, (0.96)$	$0.82_{-0.28}^{+0.09}, (0.95)$	$0.79_{-0.31}^{+0.11}, (0.96)$	$0.82_{-0.29}^{+0.09}, (0.95)$
Dual D	$0.80_{-0.29}^{+0.10}, (0.95)$	$0.84_{-0.24}^{+0.08}, (0.94)$	$0.82_{-0.29}^{+0.11}, (0.96)$	$0.85_{-0.24}^{+0.08}, (0.94)$

The comparison is given for the two-stage models and the different flavors of dual-segmentation heads models. For a description of the metrics, see “Evaluation metrics” Section

### BEM-inference

We refine the inference step using a binary background/bone-tissue segmentation $y_{\mathrm {bg/bt}}$. This additional prediction can stem from a second head of the multi-class network, from an additional network, or from a completely different segmentation method.

In standard inference, all classes, including the background class, are predicted in one step. Instead, we use the binary prediction $y_{\mathrm {bg/bt}}$ as a guide and ignore the background class 0 in the distinct bone prediction. We split our *N* classes into one background and $N-1$ foreground classes. The final prediction is then set to be either background, if $y_{\mathrm {bg/bt}}=0$ or to the most likely foreground class.

In contrast to simple masking of the finished multi-class prediction in post-processing, which could remove false negative foreground voxels, this method addresses both false negatives and false positives. An illustration of a simplified case in 2D with two foreground classes can be found in Fig. 4.

### Connected component-based label correction

After completion of the inference process, we automatically refine the segmentation by reassigning connected components. We build upon the post-processing approach of keeping only the biggest connected component per label [11]. However, instead of assigning all smaller components to the background, we assign them to their neighboring biggest component. To do so, we define sets of bones that are easily confused by a model. Within such a set *L*, we identify all connected components per class and choose its largest connected component as the class anchor. Adjacent smaller components of other classes are then reassigned the anchor label. The sets *L* are chosen based on anatomical knowledge and on the most frequent confusions among bone classes observed on the validation set. To save-guard against very fragmented segmentations, an upper threshold *u* of connected components ensures a runtime of ${\mathcal {O}}(|L |^2u)$. Different sets can be processed in parallel to speed up the computation. We chose $u=100$ and worked with 16 sets *L*, of size $4\le |L |\le 12$. The detailed groups are shared along with the code at https://gitlab.com/cian.unibas.ch/cars2022-bem-inference.

### Evaluation metrics

As our main metric, we use the Sørensen-Dice similarity coefficient $\text {DSC}_c$ for each segmentation class *c*. To assess the overall performance of our models, we give the median, and the 16- and 84-percentile $(\sim 1 \sigma )$ of all classes where at least one true-positive voxel has been predicted as $\mathrm {median}_{-\sigma }^{+\sigma }$. We account for the remaining classes, those with $\mathrm {DSC_c}=0$, by providing the fraction of classes where $\mathrm {DSC_c}>0$ in brackets. We account for the completely missing classes by providing the fraction of detected classes in brackets.

## Results and discussion

Our results show how a BEM-inference combined with connected-component correcting post-processing can improve automated distinct bone segmentation from upper-body CTs. Our evaluation involves two different datasets, four flavors of U-Nets with dual segmentation heads, and a two-stage approach.

**Fig. 6:**
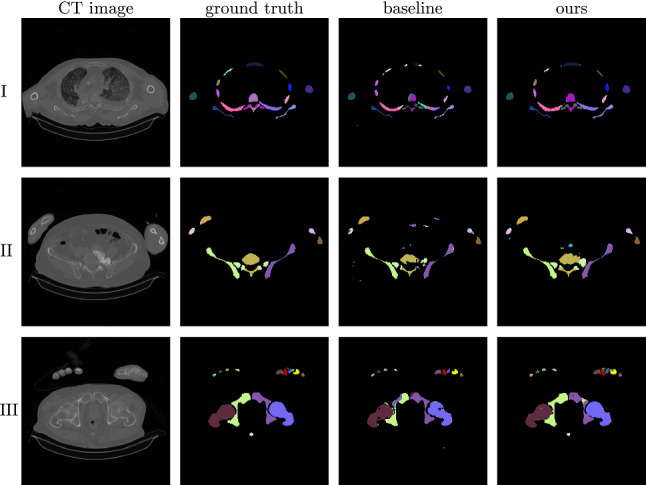
Segmentation results and typical errors obtained with the baseline U-Net model and our Dual D model with BEM-inference and post-processing. Using the baseline model, ribs are often not segmented as one, but are assigned multiple labels (I). The post-processing remedies this issue visibly. Other frequent errors occur around the border of vertebrae, especially in the presence of calcifications (II). Within big bones such as hips and femurs, we observe holes and islands where the left/right part of the label has been mixed up (III)

**Table 3:** Synthetic dataset: Results in DSC, comparing the segmentation performance when using baseline inference, against our BEM-inference, with and without label correction

Model	Baseline	+ BEM-inference
Two-stage: gt binary seg.	$0.973_{-0.240}^{+0.030}, {(1.00)}$	$0.991_{-0.250}^{+0.010}, {(1.00)}$
Dual A: parallel losses	$0.970_{-0.230}^{+0.030}, {(1.00)}$	$0.970_{-0.230}^{+0.030}, {(1.00)}$
Dual B: parallel final layers	$0.971_{-0.230}^{+0.030}, {(0.99)}$	$0.978_{-0.230}^{+0.020}, {(0.99)}$
Dual C: sequential heads	$0.963_{-0.260}^{+0.040}, {(0.99)}$	$0.966_{-0.250}^{+0.030}, {(0.99)}$
Dual D: separate decoders	$0.975_{-0.230}^{+0.020}, {(1.00)}$	$0.982_{-0.230}^{+0.020}, {(1.00)}$

The comparison is given for the two-stage models and the different flavors of dual-segmentation heads models. For a description of the metrics, see “Evaluation metrics” Section

**Table 4:** Comparison to other published work on distinct bone segmentation

	Ours (median)	[11] (median)	[2] (mean)
L3	0.85	0.85	0.91
Sacrum	0.90	0.88	
Clavicula	0.92		0.57
Hamate	0.86		
Inference time per scan (min)	$\sim 5$		$\sim 20$
Scans in dataset (#)	11	100	19
Classes (#)	126	49	62

Results in DSC

Test We evaluated the errors most commonly experienced while conducting a baseline U-Net segmentation on our upper-body CT dataset. The confusion matrix (Fig. 5, left, first column) illustrates our finding, that many errors originate from predicting bones as background, as opposed to confusing one bone for another. This type of error is reduced when using our proposed methods (Fig. 5, right, first column).

We conducted an ablation study on the upper-body CT dataset, where we examined the influence of how the binary prediction was created (two-stage versus networks with dual segmentation heads), the network architecture, and the label correction post-processing. The results are listed in Table 2. Common errors are illustrated in Fig. 6. The proposed method using a Dual D model, BEM-inference and the post-processing label correction detected correct voxels in 94% of all bones and achieved a median DSC of 0.85, which is an improvement over our baseline with a median of 0.78. Both the BEM-inference and post-processing contribute individually to the improved DSC scores, but the strongest results are achieved in combination.

We observe a small increase of the fraction of bone classes with $\mathrm {DSC>0}$ when using the enhanced inference, and a slight decrease when using the post-processing. The majority of classes with a DSC of 0 are small bones located in the hands.

In Table 4, we compare our results to the hierarchical atlas segmentation by Fu et al. [2] and the convolutional neural networks by Lindgren Belal et al. [11]. Our results compete well, although the use of different datasets hampers a direct comparison.

Among the models with two segmentation heads, the most complex version Dual D with two separate decoders led to the best results. Merely training two decoders simultaneously on two different loss functions led to first improvements over our baseline, which improved even further when using BEM-inference and label-correction.

The results of the two-stage approach depend on the performance of both the multi-class and binary segmentation model. We used a binary segmentation predicted by the baseline 3D U-Net trained on the background/bone-tissue segmentation task. This network achieved a mean DSC of 0.94 for the binary prediction, which is in the range of results reported in [5] and [6]. For comparison, we used the binary ground truth data during the BEM-inference step to get an upper bound of how much improvement was possible. We observed a steep improvement of the results, suggesting that the investment into a good binary segmentation clearly pays off. Since the manual labelling of the ground truth data is less time-consuming and cumbersome for the binary segmentation as opposed to a full multi-class segmentation, the additional binary labelling of new training data might yield a good return on investment.

In comparison, the two-stage approach tends to be more troublesome than a dual head architecture since it involves the training and tuning of two networks and a sequential inference first using the binary network, then the multi-class network. The use of a network with two segmentation heads simplifies this task to training one network only and performing an end-to-end inference. If additional scans with binary ground truth labelling are available, they can be used to fine-tune the binary segmentation head.

There is currently no public upper-body CT dataset with complete distinct bone labelling available and our in-house dataset cannot be shared as of yet. Therefore, we provided additional results on our public synthetic dataset. The results on the synthetic dataset mirror the findings in the upper-body dataset. BEM-inference improves the segmentation both for the two-stage approach and the architectures with dual segmentation heads (see Table 3 and Fig. 2).

## Conclusion

We proposed BEM-inference to improve the automated segmentation of distinct bones from upper-body CT scans. A substantial part of the segmentation errors made by 3D U-Nets does not originate from the mixing-up of different bone classes but from the mistaking of background for the foreground , and vice versa. Therefore, we proposed an inference method that uses the information gained in a binary background/bone-tissue segmentation to improve upon the multi-class inference. We compared two approaches to obtain the necessary binary segmentation: (1) Networks with dual segmentation heads that are trained on both tasks simultaneously, (2) and a two-stage approach where separate networks are trained for the multi-class and the binary segmentation task. Using our proposed inference lead to improvements on all architectures and on both datasets, with and without our label-correction post-processing . The class-median DSC of the dual decoder network with both post-processing and BEM-inference is 0.85 on the upper-body CT dataset, outperforming the baseline 3D U-Net and previously reported results by other groups.

Our proposed BEM-inference is most suitable for tasks where the binary task is simpler to solve or binary labelled data is easier to obtain than the full multi-class labelled data. Since an existing multi-class ground truth segmentation can easily be converted to a binary ground truth segmentation, any multi-class model can be retrofitted to use two-stage BEM-inference. if a source of binary segmentations is available or trainable This makes BEM-inference a versatile addition to anatomical multi-class segmentation workflows.

## Data Availability

The upper-body CT dataset is not publicly available. The synthetic dataset can be recreated from code.

## Code availability

Code for post-processing and to generate the synthetic dataset is shared at: https://gitlab.com/cian.unibas.ch/cars2022-bem-inference.
